# Derivation of a Three Biomarker Panel to Improve Diagnosis in Patients with Mild Traumatic Brain Injury

**DOI:** 10.3389/fneur.2017.00641

**Published:** 2017-11-30

**Authors:** W. Frank Peacock, Timothy E. Van Meter, Nazanin Mirshahi, Kyle Ferber, Robert Gerwien, Vani Rao, Haris Iqbal Sair, Ramon Diaz-Arrastia, Frederick K. Korley

**Affiliations:** ^1^Department of Emergency Medicine, Ben Taub Hospital, Houston, TX, United States; ^2^Program for Neurological Diseases, ImmunArray, Inc., Richmond, VA, United States; ^3^Gerwien Statistical Consulting, Newington, CT, United States; ^4^Department of Psychiatry and Behavioral Science, Johns Hopkins Bayview Medical Center, Baltimore, MD, United States; ^5^Department of Radiology, Johns Hopkins University, Baltimore, MD, United States; ^6^Department of Neurology, Perelman School of Medicine, University of Pennsylvania, Penn Presbyterian Medical Center, Philadelphia, PA, United States; ^7^Department of Emergency Medicine, University of Michigan Medical School, Ann Arbor, MI, United States

**Keywords:** mild brain injury, mild TBI, biomarker, machine learning, neurogranin, neuron-specific enolase, metallothionein 3

## Abstract

**Background:**

Nearly 5 million emergency department (ED) visits for head injury occur each year in the United States, of which <10% of patients show abnormal computed tomography (CT) findings. CT negative patients frequently suffer protracted somatic, behavioral, and neurocognitive dysfunction. Our goal was to evaluate biomarkers to identify mild TBI (mTBI) in patients with suspected head injury.

**Methods:**

An observational ED study of head-injured and control patients was conducted at Johns Hopkins University (HeadSMART). Head CT was obtained (ACEP criteria) in patients with Glasgow Coma Scale scores of 13–15 and aged 18–80. Three candidate biomarker proteins, neurogranin (NRGN), neuron-specific enolase (NSE), and metallothionein 3 (MT3), were evaluated by immunoassay (samples <24 h from injury). American Congress of Rehabilitation Medicine (ACRM) criteria were used for diagnosis of mTBI patients for model building. Univariate analysis, logistic regression, and random forest (RF) algorithms were used for data analysis in R. Overall, 662 patients were studied. Statistical models were built using 328 healthy controls and 179 mTBI patients.

**Results:**

Median time from injury was 5.9 h (IQR, 4.0; range 0.8–24 h). mTBI patients had elevated NSE, but decreased MT3 versus controls (*p* < 0.01 for each). NRGN was also elevated but within 2–6 h after injury. In the derivation set, the best model to distinguish mTBI from healthy controls used three markers, age, and sex as covariates (C-statistic = 0.91, sensitivity 98%, specificity 72%). Panel test accuracy was validated with the 155 remaining ACRM+ mTBI patients. Applying the RF model to the ACRM+ mTBI validation set resulted in 78% correctly classified as mTBI (119/153). CT positive and CT negative validation subsets were 91% and 75% correctly classified. In samples taken <2 h from injury, 100% (10/10) samples classified correctly, indicating that hyperacute testing is possible with these biomarker assays. The model accuracy varied from 72–100% overall, and had greater accuracy with increasing severity, as shown by comparing CT+ with CT− (91% versus 75%), and Injury Severity Score ≥16 versus <16 (88% versus 72%, respectively). Objective blood tests, detecting NRGN, NSE, and MT3, can be used to identify mTBI, irrespective of neuroimaging findings.

## Introduction

There are nearly 5 million annual visits to the emergency departments (EDs) in the US alone for evaluation of head injuries (1, 2). An estimated 70–90% of these are subsequently classified as mild traumatic brain injury [mild TBI (mTBI); Glasgow Coma Scale (GCS) = 13–15 (3)], a population in which diagnosis can be challenging due to the heterogeneous nature of the disorder (4, 5). In the acute setting, neuroimaging techniques are commonly used to evaluate patients with suspected TBI. The decision to obtain cranial computed tomography (CT) scans is guided by the American College of Emergency Physicians criteria, and the Canadian Head CT Rule (6, 7). Of patients receiving a head CT for trauma, over 90% will have no anatomic abnormality. However, it is recognized that while CT is sensitive to pathologies such as intracerebral hemorrhage, it is insensitive to diffuse axonal injury (8), which is a predominant pathology after TBI (9). Recent studies with acute magnetic resonance imaging (MRI) find that approximately 25–40% of CT negative patients have trauma-related abnormalities noted on MRI (10–12).

The American Congress of Rehabilitation Medicine (ACRM) defines mTBI as an acute injury resulting from mechanical force impacting the head, associated with an initial GCS score of 13–15 after 30 min, and any of loss of consciousness (LOC) <30 min, posttraumatic amnesia <24 h, a period of confusion at the time of the accident (feeling dazed, disoriented, confused), or other transient neurologic abnormalities such as focal signs or seizures (13). One limitation of the ACRM definition is the subjectivity of some of the criteria used in assessment. For example, “feeling dazed, confused, and disoriented” is vague and often difficult to ascertain. Such subjective reports are nonspecific, and are confounded by emotional and psychological factors, and are problematic to accurately assess in the presence of intoxication with alcohol or other psychoactive substances (14, 15). Because of these inconsistencies, the reliability of the ACRM criteria and their usefulness as a guideline for treatment decisions is limited. Derivation of an objective diagnostic test using blood-based biomarkers could provide more reliable identification of mTBI in any acute care setting.

There is currently no pharmacologic post-TBI intervention that is effective in altering the natural course of recovery following a head injury. It is clear, however, that additional trauma after the index injury increases the risk of adverse events and should be avoided (16, 17). As the decision to permit return to an environment with a high probability of TBI re-exposure is subjective and fraught with conflicting influences, identifying those at risk for serious adverse consequences is an important clinical challenge. An objective mTBI test would provide guidance as to the prudence of allowing a patient to return to an environment at risk for TBI.

Another potential advantage of an objective mTBI test relates to the heterogeneous nature of the TBI population. mTBI patients may have a course that ranges from asymptomatic to significant disability, with symptoms emerging weeks to months after the initial evaluation (18, 19). Not only does this impact the follow-up recommendations at the initial visit but also makes it extremely difficult to evaluate the success of therapeutic interventions, as effect sizes cannot be accurately determined. The ability to identify and characterize mTBI in initially asymptomatic patients when planning investigational therapeutic studies would be of great benefit.

Several biomarkers have been studied for their utility in detecting TBI; notably, the pro-inflammatory cytokines, astroglial and neuronal proteins, and MRI evidence of neural injury. The astroglial markers Glial Fibrillary Acidic Protein (GFAP) and the calcium binding protein S100B have been studied extensively over the past three decades, with published studies suggesting both their utility and limitations. While S100B has been adopted as a guideline biomarker for TBI in the US (ACEP) and Europe, is being used as a clinical test in some EDs, and is abundantly expressed in astrocytes, it is not specific to the brain, limiting the utility in cases of polytrauma (20). Most of the published literature indicates that S100B decreases after injury, and it has been suggested that increased levels due to polytrauma are most affected during the first 48 h, after which a clearer picture of residual TBI-related levels can be obtained (21). S100B has been shown to be elevated in moderate to severe TBI and to correlate with secondary injury and poor outcome. In mTBI, levels generally decrease to normal with the first 12 h (21). Therefore, S100B has support as a useful marker within these contexts of use. GFAP is an abundant intermediate filament protein specific to astroglia as well, but is not sensitive or specific in mild injury such as sports-related concussion. Both S100B and GFAP have been shown to be sensitive and informative biomarkers in moderate to severe TBI, however, and correlate with inflammation and hemorrhage, respectively (22, 23). Several studies have looked at the prognostic value of these markers.

A number of neuron-specific serum and CSF proteins have also been reported to be elevated in head-injured patients diagnosed with TBI, including, most notably, brain-derived neurotrophic factor (BDNF) (1, 2), neurofilament light chain [NF-L (24)] and neurofilament heavy chain [NF-H (25)], Tau and phosphorylated Tau [pTau (26)], neuron-specific enolase [NSE (27)], and ubiquitin carboxyterminal hydrolase like 1 [UCHL1 (28)]. Each of these is predominantly expressed in neurons and is localized in different areas of the neuronal infrastructure, including axonal localization (NF-L, NF-H, Tau), extracellular (BDNF), and cytoplasmic (NSE and UCHL1). Each of these proteins has also been shown to have some utility as TBI biomarkers in certain contexts of use. These neuronal markers need further development to better understand their utility and specificity in different clinical contexts and to determine which markers can best distinguish mTBI from non-head-injured individuals in the acute setting. Other important elements to consider in evaluating candidate blood biomarkers is that detection is dependent on the dynamic changes in protein clearance from CSF to blood and the underlying biology of each biomarker protein after injury (e.g., binding to receptors or other proteins). Not all biomarkers are reliably detectable in the first few hours after injury, with rates of change of protein levels and resolution to normal levels differing considerably between biomarkers and individuals (21).

Small neuronal proteins may more easily leak out of damaged plasma membranes and be detectable earlier than proteins tied to the axonal infrastructure. Two such proteins, neurogranin (NRGN, 15 kDa) and metallothionein 3 (MT3, 7 kDa) have been under evaluation in our laboratories for this purpose. Both proteins have some implication in chronic neurodegenerative disease pathobiology, could serve as early markers of TBI useful in diagnosis, and could play a role in long-term monitoring if shown to play a role in neurodegenerative changes after TBI (29, 30). A recently published study from our group suggests that NRGN is a novel marker that is elevated in TBI, and other reported studies indicate that it may be involved in memory function, since it is known to play a role in post-synaptic signaling in events such as long-term potentiation and has been shown to be both expressed in hippocampal neurons and essential in memory consolidation in rodent models (31). MT3 is a neuronal member of the metallothionein family of proteins that regulate the bioavailability of metal ions, such as copper, cadmium, and zinc (32). MT3 has been shown to increase in expression during brain development and to reach its highest level in mature post-natal neurons. In animal models of neurodegeneration, MT3 protein has been shown to bind to neurofibrillary tangles, amyloid, and Synuclein alpha aggregates and to sequester copper, insulating the microenvironment from free metal-associated toxicity (33, 34). MT3 may, therefore, have a neuroprotective role after TBI and, therefore, play a role in patient recovery.

Our purpose was to test the utility of the novel, small molecular weight TBI biomarkers MT3 and NRGN, together with a cytoplasmic neuronal protein that has already been shown to correlate with disease severity, NSE (35, 36). This multi-analyte panel of three neural biomarkers, detectable in blood, should be less dependent on significant proteolysis related to cell damage and cell loss and, therefore, reflective of more subtle injury and increased permeability of neuronal membranes due to cellular damage. The study was designed to evaluate the three biomarkers individually and in multi-analyte panels for their usefulness in objectively identifying mTBI, irrespective of CT findings or clinical symptoms. The establishment of such a test would allow for objective screening for mTBI in any point of care setting.

## Materials and Methods

### Enrollment of Subjects

**Figure 1:**
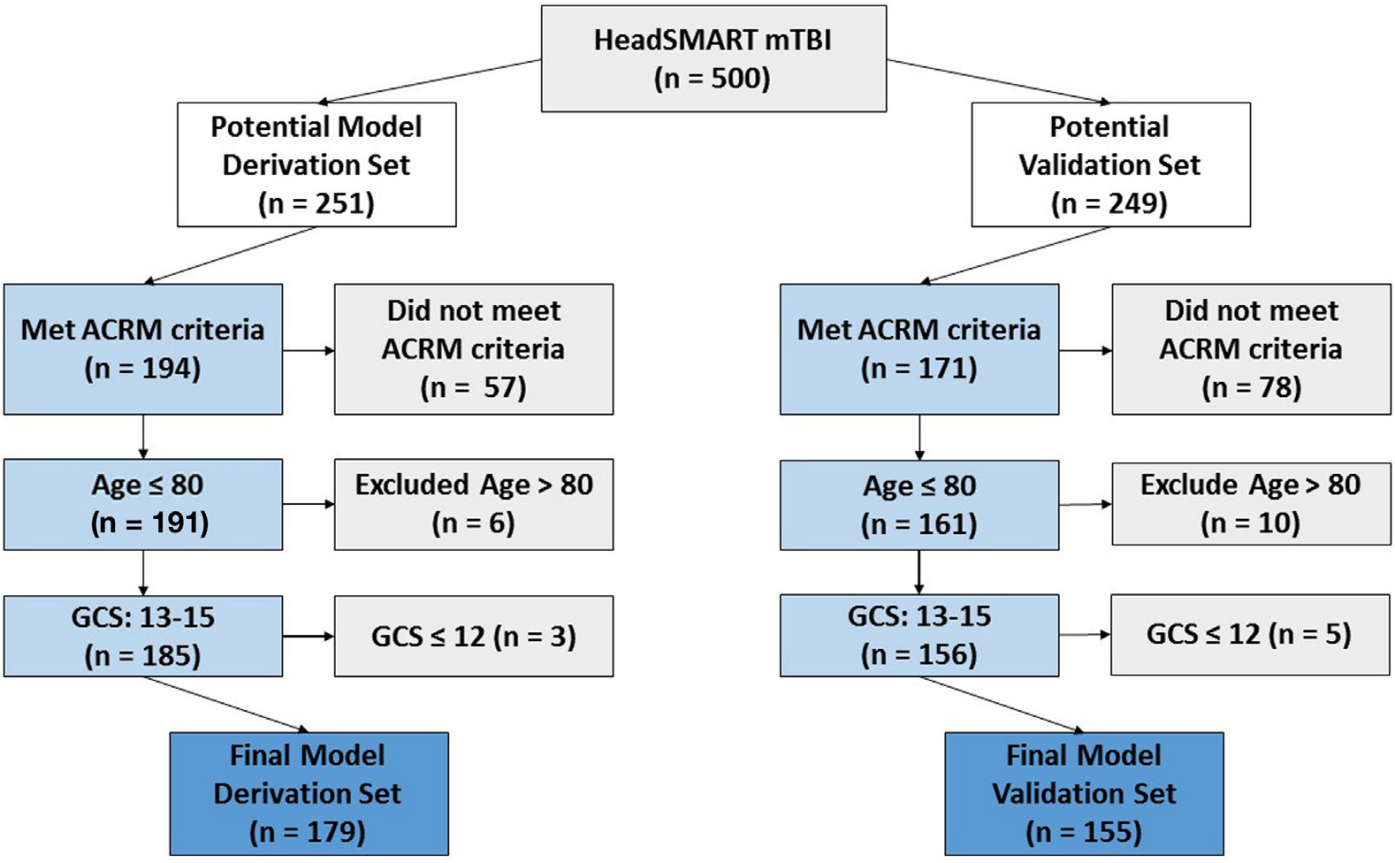
Flow diagram demonstrating the criteria for HeadSMART mild TBI (mTBI) patient selection and effect on sample size for the model derivation and validation sets. Final model set numbers also reflect the removal of a small number of samples that did not pass quality control in repeated assay runs.

Patients included in this analysis were evaluated for a blunt head injury at two EDs within the Johns Hopkins University Hospital System (Baltimore, MD, USA) and enrolled in the Head Injury Serum Markers for Assessing Response to Trauma (HeadSMART) study. The study was a prospective observational study enrolled for the purposes of biomarker development for TBI diagnosis and monitoring. The enrollment period was from 2014 to 2017. Eligibility criteria included being 18–80 years of age, providing written informed consent, having been eligible and received a head CT scan, and having a GCS of ≥13. Of the 500 enrolled patients, 8 were excluded because of the GCS value of less than 13 and 30 patients were excluded due to advanced age (>80, which were not used due to being beyond the range of the approved protocol). One sample was removed because of both age and GCS (age = 88, GCS = 11). Patients with an initial blood sample collected after the first 24 h of injury were not examined in this HeadSMART study. Patients in the TBI cohort received a standard of care head CT per the ACEP criteria for TBI imaging as part of the ED workup and were assessed by ACRM criteria. The patient data collected by physicians and trained research staff included demographics, past medical history, signs and symptoms following injury, clinician interview, mechanism of injury, physical findings, social history, and detailed contact information. The HeadSMART patients were divided evenly to provide a model derivation (*n* = 251) and validation set (*n* = 249). To ensure that model building and testing was performed using mTBI, only the patients meeting ACRM criteria for mTBI diagnosis were used (see Figure 1 for the study outline). From the 500 hundred patient HeadSMART study 179/251 were ACRM+, used for model derivation, and 155/249 were used for validation of the models. The flow diagram in Figure 1 shows the breakdown of patients and the selection process for the training and testing the models. No difference was observed in clinical data or demographics between the two cohorts. Patients were also evaluated for injury severity scores (ISS) and adjusted injury severity scores for the head and periphery, and CT images were read by a neuroradiologist for assessment of abnormal intracranial findings and skull fractures.

Two control cohorts were used for this study. In the HeadSMART study, healthy controls were enrolled at Johns Hopkins University Hospitals (Downtown and Bayview campuses, Baltimore, MD, USA; *n* = 59). The protocol required controls to have no acute medical complaints/active illness and were not ED patients. To be included, subjects had to have no known prior or active diagnosed psychiatric or neurologic disease, no history of kidney failure, stroke, brain tumor, or intracranial surgery, no known active medical conditions other than diabetes, hypertension and high cholesterol, no recent blood transfusion, not pregnant, no recreational drug use within 2 weeks of blood draw, and only included non-smokers with a blood pressure below 140/80. Further details about the design of HeadSMART have been published elsewhere (37).

To increase the number of healthy controls, a second heathy control cohort was obtained at Baylor College of Medicine (BCM, Houston, TX, USA; *n* = 269), and consisted of non-patient ED waiting room volunteers enrolled after providing informed consent. Comprehensive health histories were taken to exclude head injury within 6 months, and they had no known neurological disease, cancer, or other major illness. All samples were processed with standardized protocols. Only samples with available clinical data were used. Institutional Review Board approval was obtained from all institutions.

All TBI blood samples were obtained in the ED by research staff within 24 h of injury. From both TBI and controls, 10 cc of whole blood was drawn, separated in serum collection tubes (Vacutainer, Becton Dickenson; Durham, NC, USA), de-identified, processed and stored at −80°C. The samples were then shipped on dry ice to the ImmunArray lab (Richmond, VA, USA) for testing. Visual inspection was used to screen for hemolysis in test samples, and four samples were removed from the analysis, on the basis of having evidence of hemolysis.

### Biomarker Assays

Serum levels of NRGN, NSE, and MT3 were tested using a sandwich immunoassay with electrochemiluminescence detection on a Quickplex 120 plate reader (Mesoscale Discovery; Rockville, MD, USA). Recombinant full-length human NRGN and NSE proteins (Origene Technologies, Inc., Rockville, MD, USA) and recombinant full-length human MT3 (NovoPro Biosciences, Shanghai, China) were used to generate a standard curve relating analyte concentration to luminescent signal. Mouse monoclonal capture antibodies for MT3 and NRGN, and rabbit polyclonal antibodies for NRGN were produced by ImmunArray (ImmunArray USA, Inc.; Richmond, VA, USA). Other antibodies were obtained from commercial sources for MT3 (rabbit polyclonal antibody; NovoPro Biosciences, Shanghai, China) and NSE (R&D Systems; Minneapolis, MN, USA). Samples were tested in duplicate wells in replicate assays and the average concentrations obtained *via* 4PL regression curve equation from the standard curve. Acceptance criteria included replicate samples varying less than 10% (CV), percent recovery of 80–120% and regression curve linearity above 0.99. The lower limit of detection (LLOD) for NRGN, NSE, and MT3 are 0.041, 0.033, and 0.018 ng/ml, respectively.

### Statistical Analysis

Descriptive statistics were calculated for clinical features and biomarker data, assessing means and SDs for continuous variables, and counts and percentages for categorical variables. Biomarker values below the LLOD were substituted with a randomly generated number between 0 and 0.5 times the LLOD for that biomarker assay, consistent with published standards (38). The biomarker concentrations were transformed using the logarithm with base 2 to reduce skewness in the distributions. Kruskal–Wallis tests were used to determine significant changes in biomarkers over time (between time points, α = 0.05), and univariate analysis in logistic regression (LR) was performed to test for significant elevation (NSE, NRGN) or decrease (MT3) compared to the distribution of the healthy control population (*n* = 328, α = 0.05).

Performance of single and multi-marker combinations was compared using C-statistics. For modeling, patients with missing biomarker data (samples not evaluated) were excluded. For each panel, a LR model was fit and the C-statistic was estimated *via* stratified 10-fold cross-validation (39, 40). Models were also constructed with a panel of all biomarkers using the random forest (RF) algorithm, and performance re-assessed using stratified 10-fold cross-validation. To further test the accuracy of the model, the best RF model was applied to the remaining 155 ACRM+ patients from the HeadSMART cohort that was not used in model building. The model was also tested on a subset of the HeadSMART test samples with the blood draw time less than 2 h (*n* = 10), to examine the utility of the model in the earliest period post-injury (hyperacute).

Clinical utility was assessed by defining model performance threshold that provided a sensitivity of greater than 98% for an ACRM positive diagnosis. All data were analyzed by the statistical programming environment R version 3.3.0 and the integrated development environment for R, RStudio version 1.0.136 (41).

## Results

Overall, 662 patients were utilized in the study. For model development, a derivation set of 507 samples was used, where 179 were mTBI (ACRM+). The median time from injury to ED presentation was 5.9 h (IQR, 4.0; range 0.8–24 h). While the sex distribution was similar between the HeadSMART and BCM healthy control populations (univariate analysis in LR, *p* = 0.46), there were more females (56.7% females) in HeadSMART and more males in the BCM control group (34.4% females). Demographics, clinical features, and mean biomarker levels for healthy and mTBI cohorts are reported in Table 1. The clinical and demographic data for HeadSMART mTBI patients resemble those reported for other published cohorts (37).

**Table 1:** Demographics, acute clinical symptoms, mechanisms of injury and biomarker concentrations in TBI patients and healthy control subjects used in the study.

	Healthy control			Head injured		
	Baylor (*n* = 269)	HeadSMART (*n* = 59)	Total healthy (*n* = 328)	ACRM+ derivation (*n* = 179)	ACRM+ validation (*n* = 155)	Total mild TBI (*n* = 334)
Mean age, years (SD)	40.65 (±13.43)	31.27 (±12.27)	38.96 (±13.21)	40.40 (±17.18)	45.36 (±17.28)	42.70 (±17.20)
Male (%)	119 (44.24%)	23 (38.98%)	142 (43.29%)	123 (68.72%)	96 (61.94%)	219 (65.57%)
**Race**						
White	156 (57.99%)	28 (47.46%)	184 (56.10%)	83 (46.37%)	77 (49.68%)	160 (47.90%)
Black	89 (33.09%)	15 (25.42%)	104 (31.71%)	84 (46.93%)	69 (44.52%)	153 (45.81%)
Asian	15 (5.58%)	14 (23.73%)	29 (8.84%)	2 (1.12%)	1 (0.65%)	3 (0.90%)
Other (includes missing)	9 (3.34%)	2 (3.39%)	11 (3.35%)	10 (5.59%)	8 (5.16%)	18 (5.39%)
**Ethnicity**						
Hispanic or Latino	81 (30.11%)	3 (5.08%)	84 (25.61%)	16 (8.94%)	5 (3.23%)	21 (6.29%)
Not Hispanic or Latino	188 (69.89%)	56 (94.92%)	244 (74.39%)	163 (91.06%)	150 (96.77%)	313 (93.71%)

**TBI Patients Only**						

**Mechanism of injury**						
Struck by motor vehicle				19 (10.61%)	17 (10.97%)	36 (10.78%)
Motor vehicle collision				50 (27.93%)	37 (23.87%)	87 (26.05%)
Fall >3 ft or >5 stairs				19 (10.61%)	20 (12.90%)	39 (11.68%)
Other fall				23 (12.85%)	36 (23.23%)	59 (17.66%)
Assault				37 (20.67%)	26 (16.77%)	63 (18.86%)
Struck by/against object				7 (3.91%)	9 (5.81%)	16 (4.79%)
Pedal cycle without helmet				2 (1.12%)	3 (1.94%)	5 (1.50%)
Motorcycle				19 (10.61%)	7 (4.52%)	26 (7.78%)
Other				3 (1.68%)	0 (0.00%)	3 (0.90%)
Computed tomography positive				41 (22.91%)	23 (14.84%)	64 (19.16%)
**Glasgow Coma Scale**						
13				3 (1.68%)	3 (1.94%)	6 (2.69%)
14				29 (16.20%)	21 (13.55%)	50 (14.97%)
15				147 (82.12%)	131 (84.52%)	278 (83.23%)
Altered mental status				120 (67.04%)	109 (70.32%)	229 (68.56%)
Amnesia				129 (72.07%)	118 (76.13%)	247 (73.95%)
Depression				58 (32.40%)	47 (30.32%)	105 (31.44%)
Loss of consciousness				137 (76.54%)	119 (76.77%)	256 (76.65%)
Severe headache				101 (56.42%)	91 (58.71%)	192 (57.49%)
**Serum biomarker protein concentration (ng/ml)**						
Mean (SD) Neuron-specific enolase (NSE)	3.85 (±5.74)	1.85 (±1.47)	3.48 (±5.28)	12.22 (±37.88)	8.89 (±12.36)	10.68 (±28.99)
Mean (SD) neurogranin (NRGN)	14.08 (±29.82)	18.62 (±32.15)	14.93 (±30.27)	11.53 (±22.50)	13.41 (±33.51)	12.39 (±28.08)
Mean (SD) metallothionein 3 (MT3)	0.84 (±5.53)	0.07 (±0.08)	0.51 (±4.18)	0.12 (±0.35)	0.14 (±0.42)	0.13 (±0.38)
**Univariate ***P***-value (each group versus total healthy control)**						
NSE				<0.01	<0.01	<0.01
NRGN				0.05	<0.01	<0.01
MT3				<0.01	0.08	<0.01

**Figure 2:**
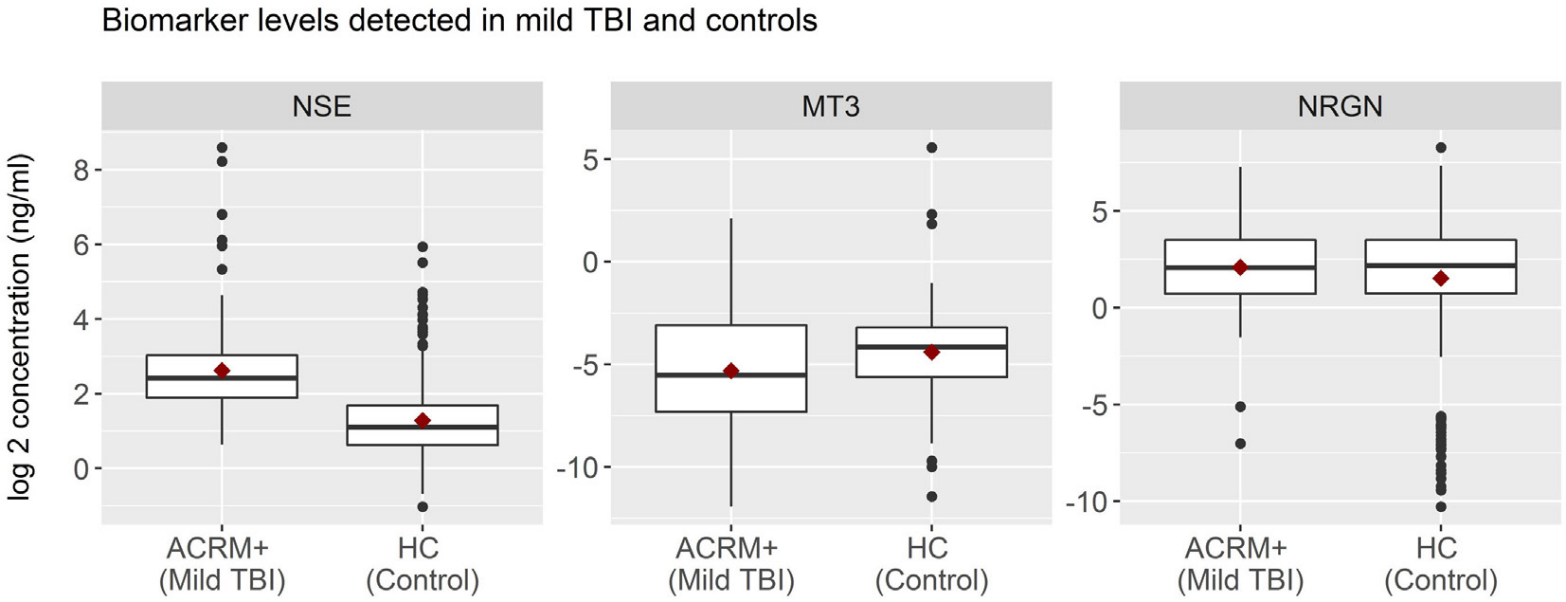
Distribution of biomarkers in mild TBI (mTBI) and healthy control subjects. Distribution of biomarker concentrations, analyzed according to TBI status. For each protein marker assay, box plots show log2-transformed detected serum levels for ACRM+ mTBI patients (left side of each subplot) versus healthy control subjects (right side of each subplot). The horizontal black lines represent the median of each distribution; red diamonds indicate the mean for the subgroup. Black dots are individual patients with values 1.5 times beyond the interquartile range and are, therefore, outliers.

**Figure 3:**
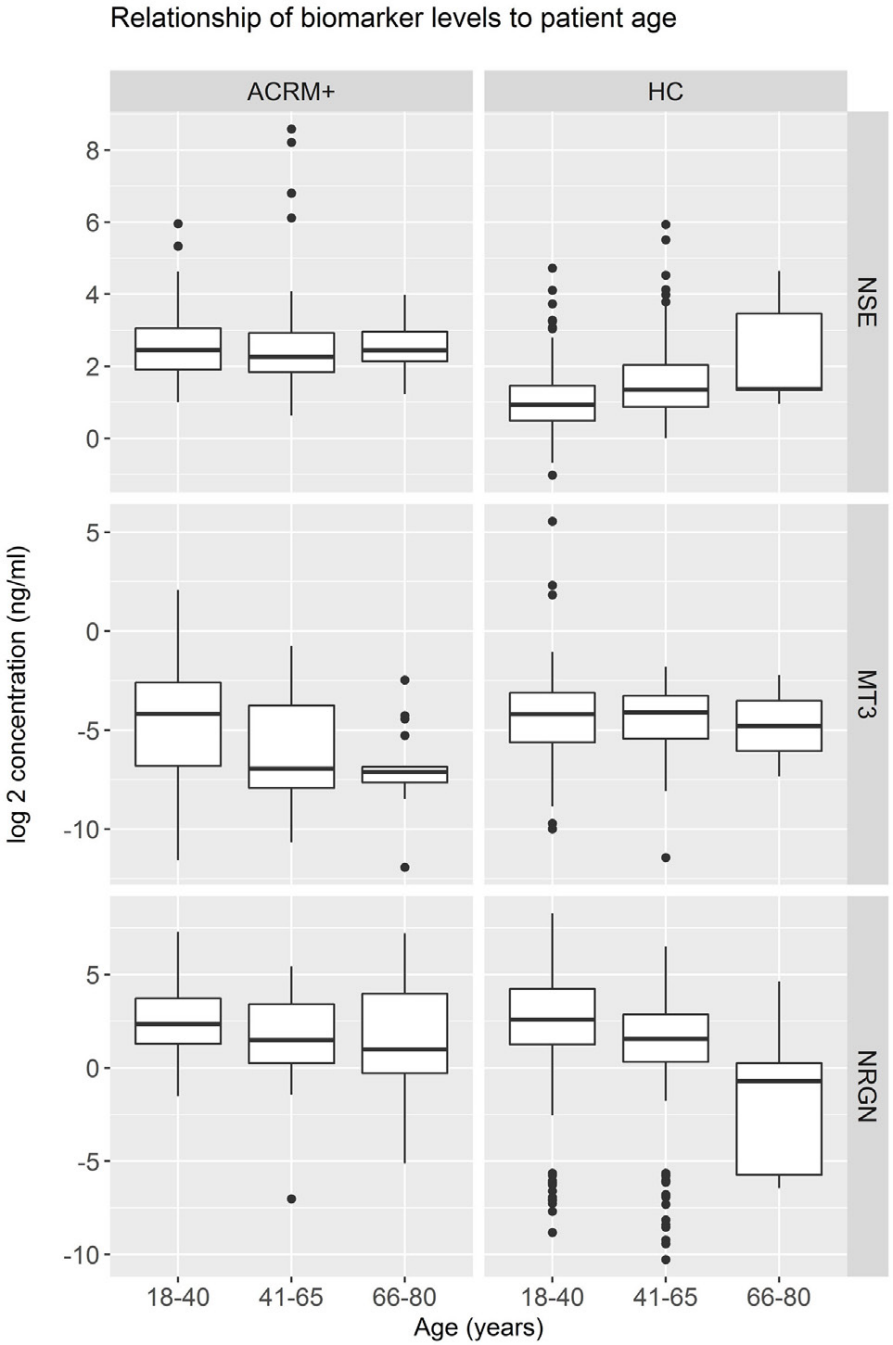
Distribution of biomarkers in mild TBI (mTBI) ACRM+ and healthy control patients among different age groups. Distribution of biomarker concentrations in young, middle-aged, and older adults, analyzed separately for mTBI and the general population (328 healthy controls). For each protein marker assay, box plots show log2-transformed detected serum levels for ACRM+ mTBI patients (left side of each subplot) and healthy control subjects (right side of each subplot). The red diamonds indicate the mean for the subgroup.

General results show that head-injured patients had higher levels of NSE and lower levels of MT3, compared to healthy controls. NRGN was also elevated in a subset of patients. Figure 2 shows the distributions of biomarker levels (log2-transformed), comparing mTBI patients with healthy control patients in the samples used to derive the classifier model. The boxplots represent the data used to build the LR and RF models to discriminate between mTBI and healthy control subjects. The two healthy control populations included in the study, when examined separately, were shown to have similar distributions for the three biomarkers studied. Similarly, median biomarker concentrations were not different between the two healthy control cohorts by the Rank Sum Test (all *p* values > 0.05; data not shown). Univariate relationships between controls and mTBI patients showed significant differences for each of the three biomarkers (NSE, MT3 *p* < 0.005, and NRGN *p* = 0.055), as well as sex (*p* < 0.001), but not age (*p* = 0.301). Each biomarker was also studied in three bracketed age groups, representing young adult (18–40 years), middle-aged adult (41–65 years), and older adults (66–80), in order to assess age-related changes (Figure 3). Biomarker levels were unchanged in healthy controls for MT3, but NRGN was significantly decreased with age (Kruskal–Wallis, α = 0.05), and NSE was found to be significantly increased. In contrast, in ACRM+ mTBI patients, age-related changes were detected only in MT3, with no age-related statistically significant differences for NRGN and NSE.

**Figure 4:**
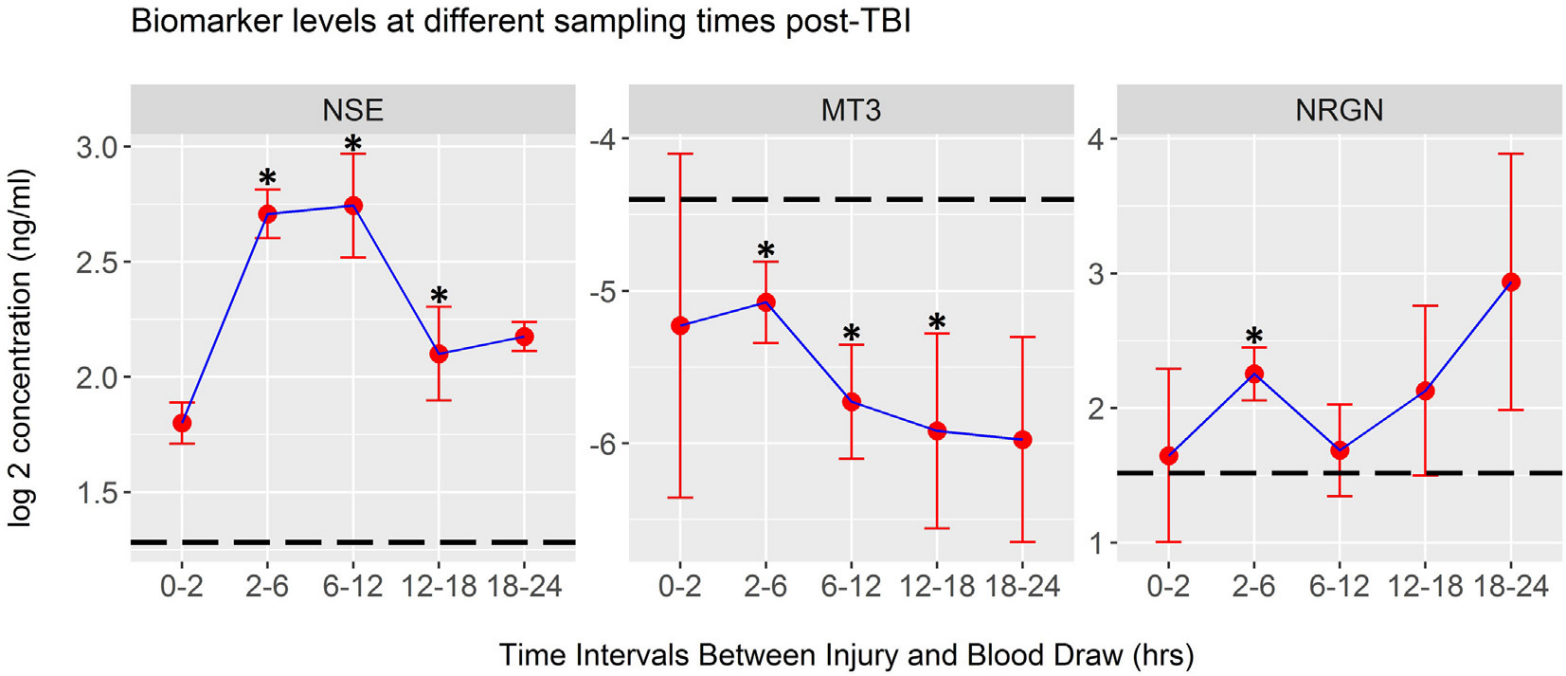
Biomarker concentrations detected at different post-injury time intervals. Plots of log2-transformed mean serum levels for ACRM+ mild TBI (mTBI) patients (red dots) are plotted with the SEM (whiskers). Significant changes were seen in neuron-specific enolase (NSE) levels between time intervals (Kruskal–Wallis Test, *p* < 0.01), whereas metallothionein 3 (MT3) and neurogranin (NRGN) were not significantly different (*p* = 0.56 and 0.63, respectively). Differences between the mean of the healthy control population and each mTBI biomarker level were tested at each time interval. Significant changes were demonstrated at multiple time points for each marker, determined by univariate analysis in logistic regression (asterisks on each plot, *p* < 0.05; dotted line, mean for 328 healthy controls; whiskers, SEM).

Mean biomarker levels were also plotted in time intervals derived from the actual elapsed time, in hours, from injury to blood draw. These data, shown in Figure 4, indicate that significant changes in biomarker levels occur over the first 24 h after injury (Kruskal–Wallis test). Univariate analysis of mTBI serum biomarker levels within each time interval, compared to healthy control levels was performed using LR. These tests showed that the mean levels of NSE, NRGN, and MT3 in serum were significantly different from controls at multiple time intervals, with NSE and NRGN increasing after injury, and MT3 decreasing after injury compared to controls (asterisks, Figure 4). Mean biomarker levels for NRGN were shown to be significantly elevated from controls 2–6 h after injury (*p* < 0.05) and to have a continued upward trend. In contrast, MT3 was found to be lower than healthy controls by 2 h after injury (*p* < 0.05), and had a continued downward trend through the first 24 h post-injury. Although MT3 levels were shown by univariate analysis to differ from healthy controls, no difference was seen between TBI subgroups with different blood draw times after injury. NSE did show significant temporal changes (*p* = 0.006), with highest detected serum levels between 2 and 12 h, whereas MT3 and NRGN were not significantly different between different blood draw time points due to heterogeneity of levels within the patients (*p* = 0.56 and 0.63, respectively).

Table 2 demonstrates the discriminative value of models built with LR using single and multiple biomarkers, in differentiating between mTBI (ACRM+) and non-injured healthy control patients. For a performance comparison, the results are presented as C-statistics (AUCs). The highest C-statistic was obtained using the combination of all three biomarkers (AUC = 0.88, sensitivity = 0.97, specificity = 0.53) to distinguish mTBI (ACRM+) from healthy controls. Increasing the panel from single markers to multiple biomarkers improved the C-statistic. NSE was the strongest performing single biomarker (AUC = 0.85), followed by MT3 (AUC = 0.59) and NRGN (AUC = 0.51). The two-biomarker combination model with NSE and MT3 performed as well as NSE, MT3, and NRGN in LR by AUC value, but the three biomarker panel had distinct advantages when tested in other model building algorithms such as RF. We also assessed whether the sex of the patient was a significant confounder in the biomarker panels that needed to be controlled for, or rather an effect modifier, in which case the panels will perform differently for males and females. Univariate analysis suggested that sex, but not age, was significant as a univariate feature. Because some age-related differences in biomarkers were found when bracketing for age groups, we included both age and sex as covariates in model building. The effect of including age and sex in model fitting was shown to be more pronounced with single markers, as indicated by improved AUC values versus biomarkers alone. Adding age and sex as covariates increased the performance of the panels by enhancing specificity (increased by 7–11% for biomarkers).

**Table 2:** Comparison of C-statistics to identify the best performing LR models that differentiate ACRM+ mTBI patients from controls using biomarkers alone, or biomarkers with patient age and sex included as covariates.

Features Included	Total (*n*)	AUC (model with biomarkers only)	AUC (model with biomarkers, age, and sex)
Neuron-specific enolase (NSE), metallothionein 3 (MT3), neurogranin (NRGN)	299	0.88	0.87
NSE, MT3	302	0.88	0.87
NSE, NRGN	483	0.86	0.85
NSE	495	0.85	0.84
MT3	306	0.59	0.66
MT3, NRGN	303	0.59	0.66
NRGN	494	0.51	0.62

Preliminary models were also generated in another machine learning algorithm, RF, to test whether additional model building techniques could improve classification. Models in RF were built using the top performing model that was obtained in the LR method (three marker panel including NSE, MT3, and NRGN), and the results compared with and without age and sex included as covariates (see Table 3). The C-Statistic in RF was 0.91, with 98% sensitivity and 72% specificity, compared with more than 20% lower specificity of the classifier in LR. The positive predictive value was improved from 75% in LR model to 84% in RF comparing three biomarkers with age and sex included as covariates. Negative predictive value improved from 93% in LR to 96% in RF. Since the highest C-statistics and other metrics were nearly equivalent between the MT3-NSE and the MT3-NSE-NRGN panels in LR, we also tested the two marker panels in RF. In contrast to LR results, the performance of the three biomarker panel gave a significant increase in specificity (from 55 to 72%) using the three biomarker panel. This improvement was seen with and without the inclusion of age and sex in the models. In general, however, age and sex increased the performance of the models.

**Table 3:** ROC curve analysis for performance of top panels in discriminating mild TBI from controls, listed by statistical method and features included.

Method	Features included	AUC	Sensitivity	Specificity	PPV	NPV
Random forest (RF)	Neurogranin (NRGN), neuron-specific enolase (NSE), metallothionein 3 (MT3), age, sex	0.91	0.98	0.72	0.84	0.96
RF	NRGN, NSE, MT3	0.91	0.98	0.71	0.83	0.96
Logistic regression (LR)	NRGN, NSE, MT3, age, sex	0.87	0.97	0.53	0.75	0.93
LR	NRGN, NSE, MT3	0.88	0.97	0.53	0.75	0.93

**Figure 5:**
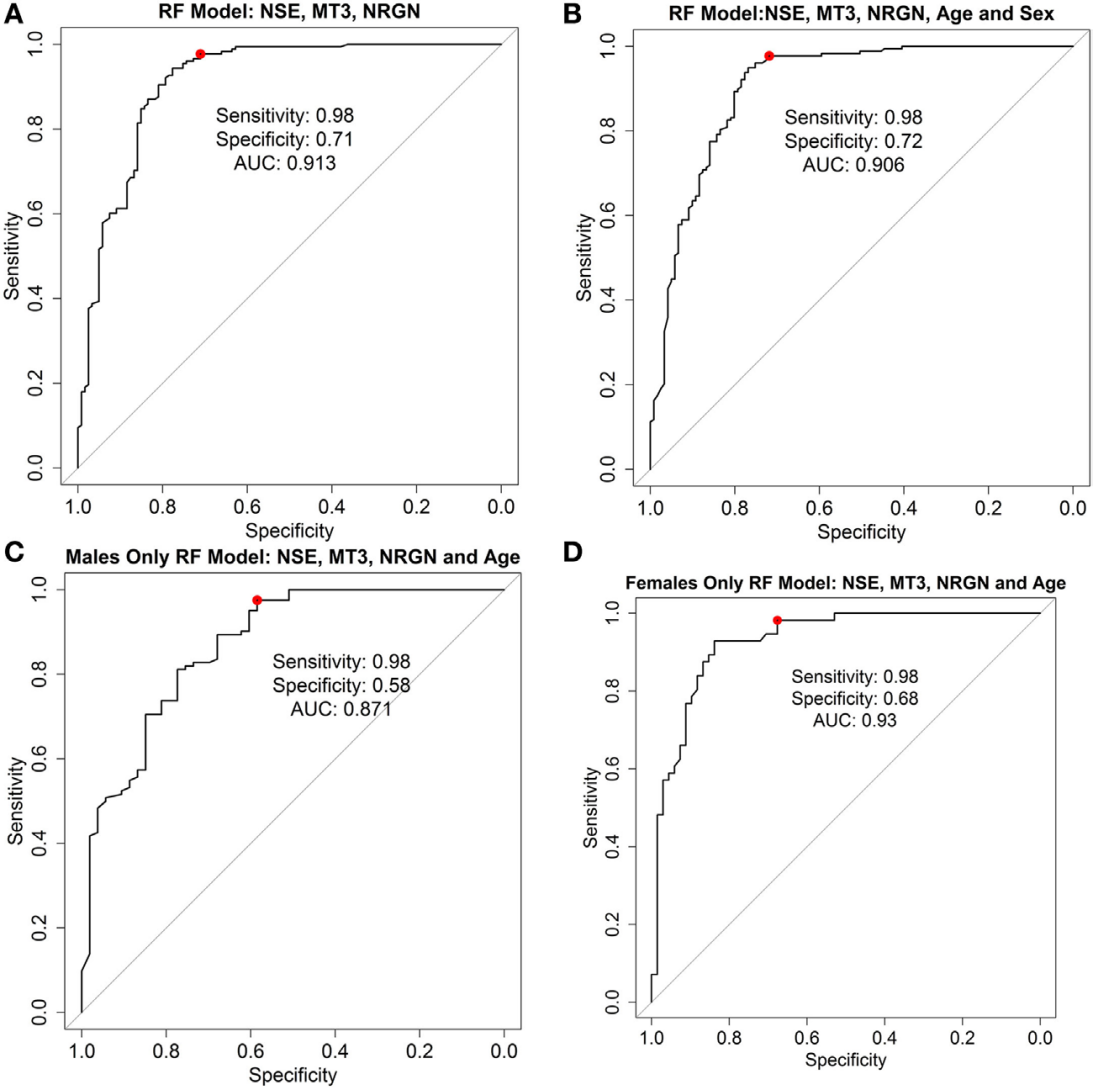
Receiver operator characteristic curves for random forest (RF) models. ROC curve analysis showing plotted models built using three biomarkers neurogranin (NRGN), metallothionein 3 (MT3), and neuron-specific enolase (NSE) only **(A)**, three biomarkers with age and sex as covariates in the model **(B)**, biomarkers and age in the male patients only **(C)**, and biomarkers and age in female patients only **(D)**. The red dot indicates the threshold point for the reported optimal sensitivity (0.98) and the corresponding specificity for classifying mild TBI versus healthy controls. Area under the curve (AUC = C-statistic; CI, confidence intervals for each AUC estimate) is indicated for each panel.

As a test to further examine the effect of sex of the patient on the model, female and male patient data were used separately to build classifier models for TBI, with age included in the models as a covariate. ROC curves for the RF models with three biomarkers alone (Figure 5A), three biomarkers with age and sex included as covariates (Figure 5B), and for male (Figure 5C) and female patients and age only (Figure 5D) are shown, and the characteristics at 98% sensitivity compared. Results in females with cross-validation were slightly greater (C-statistic 0.93, sensitivity 0.98, specificity 0.68) and male-only models slightly lower (C-statistic 0.87, sensitivity 0.98, specificity 0.51) in performance than models built with all patients together (i.e., compared with the RF model with both sexes included).

**Figure 6:**
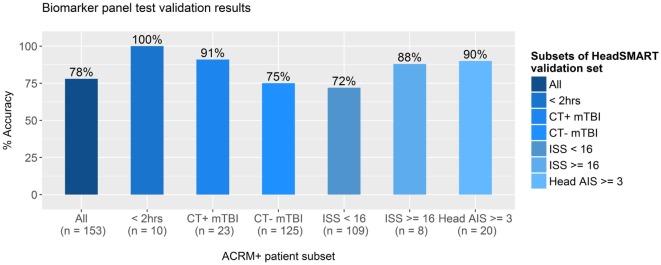
Accuracy of test results for clinically distinct subsets of TBI patients. The best performing model, fit in random forest using neurogranin (NRGN), metallothionein 3 (MT3), and neuron-specific enolase (NSE) values, patient age, and sex, was applied to subsets of mild TBI (mTBI) patients in the HeadSMART test set to test the accuracy in classification. Accuracy was assessed in the complete test set (far left bar), the subset patients that met the ACRM+ criteria, and mTBI patients with blood samples acquired less than 2 h from the index injury (<2 h) were tested with the percentage of accurate calls indicated (%). Computed tomography (CT) positive and CT negative patient subsets were also examined separately to determine the accuracy of the model. Injury severity score (ISS) was evaluated at two separate thresholds to test whether the accuracy of the model biomarker test is affected by injury severity, as well as Head AIS in the subset of patients with high Head AIS (>2) without peripheral AIS (AIS = 1 for all categories). Number of samples used to determine the accuracy for each subgroup is indicated below each bar.

To test the potential clinical utility of the derived biomarker model, additional analyses were performed by applying the model to the classification of a separate set of mTBI patients. Results for the clinical utility analysis are shown in Figure 6. The top performing model (NSE, MT3, NRGN, age, and sex in RF) was applied to the test set of the HeadSMART TBI patients, being the half of the 500 patient cohort that was not used for model derivation. This test set was analyzed for accuracy in classification by applying the RF model (NSE, NRGN, MT3, patient age, and sex) to the complete test set and to several clinically relevant subsets of the same patients. Since the model was fit to data from ACRM+ mTBI (GCS 13–15) patients in the derivation set (179 ACRM+ mTBI samples), the same criteria were used for identified mTBI in the test set population. These patients were identified as mTBI by the biomarker model with 78% accuracy (119 of the 153 patients with complete biomarker data for all three markers). To evaluate the sensitivity of the model for the earliest time points after injury, a subset of samples obtained less than 2 h from the index injury were examined for test accuracy and found to be correctly classified in 100% of individuals (10/10). CT positive patients and CT negative patient subsets were found to be correctly classified 91% (21/23 patients) and 75% (94/125) of the time, which could indicate greater sensitivity for the panel in more severe injury. The remaining five patients of the 153 had skull fracture findings by head CT but no apparent intracranial abnormalities, of which 100% were classified as mTBI by the biomarker model. Similarly, ISS was used to determine injury severity threshold, using a score of 16 or greater to indicate severe injury. In patients with total ISS of 16 or greater, the accuracy of classification by the model was found to be 88% (8/9), and in patients with lower severity of injury (15 or lower ISS), the accuracy was 72% (78/109). Because the ISS scores in TBI patients can also reflect extracranial injury, we also looked specifically at the subset of patients that had elevated Head AIS alone, with no peripheral AIS >1 and found the accuracy to be 90%. There were no patients that had peripheral injury scores higher than 1 that did not also have an elevated Head AIS in the HeadSMART cohort, but these are reflected in the total ISS severity analysis.

## Discussion

We found that NSE and NRGN were elevated, and MT3 decreased, in mTBI patients compared to controls. This is consistent with other data in the literature for all three markers (1, 2, 27, 42). The decrease detected for MT3 may be related to the sequestering of this protein at the injury site in bound protein complexes, as reported in experimental models (32). Tests of the performance of each individual marker and multi-marker panels indicated that the best discrimination between mTBI and healthy individuals was achieved using all three marker proteins. Including age and sex as covariates in model building was both necessary and improved performance, indicated by higher C-statistics, and greater specificity. The neuronal biomarker panel of NGRN, NSE, and MT3 could objectively identify mTBI patients with greater than 75% accuracy in CT negative patients. This may provide a useful test for identifying mTBI in CT negative patients. If validated in the clinical setting, then neurocognitive mTBI intervention may be a reasonable strategy (43, 44).

The usefulness of the biomarkers NRGN, NSE, and MT3 should be further evaluated in models for risk assessment, to determine whether patient stratification is possible. Such follow-on studies will require prospective evidence for any prognostic utility. In the context of use as an objective screening tool for patients presenting to the ED with a suspected mTBI, this three biomarker panel appears to identify mTBI with reasonable (72–100%) accuracy. An objective test of this type could potentially be developed to provide an indication of the severity of injury in patients. If achieved, this would be of benefit for those treated on the playing field, battlefield, or in any environment that lacks access to neuroimaging equipment. These points of care would greatly benefit from a test that could indicate which patients were in need of advanced medical services, as this information may indicate the need for immediate transport to a more comprehensive clinical setting.

The three biomarker model studied here, when controlling for age and sex bias, has good sensitivity and specificity and a high negative predictive value (96%). A preliminary assessment of clinical utility was performed by applying the internally cross-validated model to a separate validation set of patients. This analysis suggests a high sensitivity is achievable across a spectrum of mTBI subcategories (CT+, CT−, symptomatic and asymptomatic by ACRM, time from injury, etc.) and disease severities (ISS). By defining sensitivity at >98%, we identified a method to provide a reasonable screening tool for clinicians. High sensitivity in this model provides a low false negative rate, and while this is obtained at a deterioration in specificity (to only 72% in this analysis), it can ensure that the risk of a missed diagnosis is clinically unlikely. Whether it is safe to allow the patient that is negative to the test (biomarker/age/sex model) to return to activities that entail a high risk of head injury will need to be determined by further investigation and validation studies, designed to address this question.

Our study has several limitations, including the fact that the study was only performed in the ED environment and involved a limited number of centers. Thus, generalizing these findings to other non-ED environments is premature. Further, no patient decisions were made with any of our results, such that no clinical recommendations can be suggested.

An additional limitation is the fact that the healthy control population consists of a greater number of females than males and that it was in part obtained at a different location than the head-injured population. Further, the lack of a non-head-injured trauma cohort leaves the possibility of a specificity deterioration if systemic trauma has a similar biomarker effect. In general, it must also be discussed that hemolysis could interfere with the results obtained, since each of the biomarkers studied, though enriched in neurons, have also been shown to have some level of expression in other tissues including in red and white blood cells. Peripheral NSE is found in red blood cells, which may also have some level of NRGN expression, noted in recent proteomics studies throughput the body and in public databases (45). Metallothioneins are also present as circulating proteins in the blood could also contribute to detected blood levels. MT3 is mainly expressed in neurons, but public proteomic databases also show detection by mass spectroscopy in lung tissue and in the testes. We do not see differences between males and females in the TBI patients, but do see an age-related decline in some patients for MT3, as noted. Because of these possibilities, the final machine learning models have incorporated adjustments for both sex and age to adjust for these clinical differences. Such peripheral expression could affect the accurate detection of TBI-specific NSE or NRGN levels in particular during serum testing, particularly in polytrauma or hemolysis [recently reviewed for NSE in Ref. (35)]. Each of these markers, during further development and validation, will have to undergo strict testing to examine the effect of hemolysis on the model performance, and attempts made to minimize the impact of blood cell or platelet-derived protein expression on test results. How these characteristics could affect implementation of these biomarkers in a clinical setting is unclear, and further study is needed. Incorporation of a quality control feature that is sensitive to the detection of hemolysis might also be considered.

Finally, because the biomarkers selected for this investigation may not be equally present in the pediatric population, a cohort not studied in our investigation, the utility in children younger than 18 will need to be determined. Published evidence does suggest that NSE is a useful biomarker predicting neurocognitive deficits after pediatric TBI (46).

## Conclusion

The results of the study have shown that a panel of three neuronally enriched protein biomarkers, MT3, NRGN, and NSE, objectively identifies mTBI patients as compared to healthy individuals. Further studies of this biomarker panel will determine whether it can be used as a tool to stratify head-injured patients to direct and evaluate interventions. If so, this would be the first such biomarker test to be developed with high sensitivity in mTBI that is accurate across the TBI spectrum.

## Ethics Statement

This study was carried out in accordance with the recommendations of the Johns Hopkins University School of Medicine, Institutional Review Board, with written informed consent from all subjects. The protocol was approved by the Office of Human Subjects Research—Institutional Review Board. In addition, Baylor College of Medicine Institutional Review Board approved the IRB protocol for recruitment. All subjects gave written informed consent in accordance with the Declaration of Helsinki.

## Author Contributions

WP was a major contributing author and as a senior emergency medicine physician helped direct the clinical modeling. TV was the senior managing scientist leading the development and validation of the biomarker assays and data analysis and was also a lead author of the manuscript. NM was the lead data scientist who led the data analytics and developed the custom R code for the analytics and assay QC procedures. KF was a significant contributor assisting in the derivation of the R code and running biostatistics analysis. RG is a senior consulting biostatistician who reviewed all the analytical code and biostatistics to ensure there were no errors. VR was the senior Psychiatrist who assessed TBI patient symptoms and history. HS was the radiologist who reviewed and adjudicated all CT findings. RD-A was the senior neurologist and expert in biomarkers that reviewed all data and helped devise the study. FK was the principle investigator that conducted the clinical recruitment and training of additional medical staff. All authors played a role in the preparation and review of this manuscript prior to submission.

## Conflict of Interest Statement

The HeadSMART observational study was funded by ImmunArray, including support for the salaries of the research staff associated with the study. The work presented herein is the collaborative research between clinical investigators (WP, VR, HS, RD-A, and FK) and ImmunArray scientists (TV, NM, KF, RG). ImmunArray has funded this study and its employees with the intent of developing effective diagnostic tests for TBI for commercialization and eventual adoption into clinical practice. WP also has research grant funding from Abbott, ImmunArray, Janssen, Roche, and ZS Pharma, and has consulted for Bayer, Beckman, Boehrhinger-Ingelheim, Immunarray, Instrument Labs, Janssen, Relypsa, Roche, Siemens, and ZS Pharma. WP has provided expert legal testimony for Johnson and Johnson, Inc., and has ownership interest in Comprehensive Research Associates LLC, Emergencies in Medicine LLC, and Ischemia DX, LLC. Clinical investigators, however, received no compensation for this research study.
